# Supplementary material for: Association of TP53 Single Nucleotide Polymorphisms with Prostate Cancer in a Racially Diverse Cohort of Men
Source: Biomedicines. 2023 May 9;11(5):1404. doi: 10.3390/biomedicines11051404 (PMC10216554; doi:10.3390/biomedicines11051404)
Supplement: Supplementary file 1 [file biomedicines-11-01404-s001.zip › biomedicines-2372093-supplementary.pdf]

Supplementary Table S1: Description of SNPs in *TP53* gene region with MAF of at least 1%

SNP	Location	Reference	Alternate	MAF (Minor Allele Frequency)
rs144289006	intergenic	ACCT	A	0.05
rs78496430	intergenic	A	G	0.04
rs12600850	intergenic	A	G	0.56
rs62062581	intergenic	T	G	0.10
rs78337160	intergenic	CT	C	0.10
rs368098733	intergenic	AAAAAG	A	0.05
rs200145124	intergenic	GTTTA	G	0.03
rs9674772	intergenic	A	G	0.45
rs9674869	intergenic	G	A	0.03
rs150423780	intergenic	T	C	0.01
rs35119871	intergenic	C	T	0.17
rs147161837	intergenic	C	T	0.02
rs60706951	intergenic	T	C	0.11
rs72829457	intergenic	C	T	0.04
rs140032142	intergenic	G	A	0.01
rs55745760	intergenic	C	T	0.27
rs74351250	intergenic	G	A	0.02
rs144366923	intergenic	C	G	0.02
rs8073498	intergenic	A	C	0.42
rs17881045	intergenic	C	T	0.02
rs17881402	intergenic	G	A	0.11
rs17882212	intergenic	G	A	0.01
rs9893249	intergenic	T	C	0.42
rs17884512	intergenic	A	G	0.11
rs9914052	downstream	G	C	0.18
rs17881556	downstream	T	C	0.05
rs12949853	downstream	G	A	0.74
rs4968204	downstream	C	T	0.82
rs4968186	downstream	A	G	0.87
rs17883048	downstream	G	A	0.05
rs17886760	downstream	T	C	0.09
rs17887200	downstream	T	C	0.06
rs9894946	downstream	A	G	0.92
rs183153328	downstream	C	A	0.02
rs1614984	downstream	G	A	0.41
rs114831472	UTR3	G	A	0.01
rs17884306	UTR3	C	T	0.04
rs796594346	UTR3	AAC	A	0.03
rs16956880	UTR3	C	T	0.01
rs6503048	intronic	C	T	0.27
rs59098034	intronic	C	T	0.02
rs112847074	intronic	G	A	0.01
rs17883852	intronic	G	C	0.03
rs17879377	intronic	C	T	0.03
rs1641549	intronic	C	T	0.45
rs1641548	intronic	C	T	0.11
rs9891744	intronic	C	T	0.11

rs858528	intronic	C	T	0.25
rs146137066	intronic	C	T	0.01
rs1642793	intronic	T	C	0.76
rs111537395	intronic	A	T	0.01
rs12949655	intronic	A	G	0.10
rs60699444	intronic	T	A	0.20
rs75732100	intronic	C	T	0.11
rs77697176	intronic	G	A	0.02
rs1800899	intronic	A	G	0.02
rs12951053	intronic	A	C	0.10
rs12947788	intronic	G	A	0.10
rs17880604	intronic	C	G	0.01
rs539331761	intronic	A	AT	0.01
rs8069054	intronic	C	A	0.13
rs1625895	intronic	T	C	0.76
rs2909430	UTR5	C	T	0.77
rs35850753	UTR5	C	T	0.01
rs9895829	UTR5	A	G	0.10
rs1794287	intronic	A	G	0.78
**rs1042522**	**exonic**	**G**	**C**	**0.50**
**rs1800371**	**exonic**	**G**	**A**	**0.01**
rs35117667	intronic	G	A	0.02
rs17883323	intronic	G	T	0.10
rs1642785	UTR5	G	C	0.56
rs8079544	intronic	C	T	0.10
rs9894227	intronic	G	A	0.03
rs12944939	intronic	C	T	0.09

Supplementary Table S2: Literature review table for Pro47Ser

Refs	Association	AA (allele)	Population	Case/ Controls
Gnanapradeepan, K.; *et al.* [55]	+	Ser	African/ mice	
Tutton, S.; *et al.* [56]	+	Ser	African/ mice	
Pinto, G.R.; *et al.* [57]	-	Ser	Brazil	94/100
Murphy, M.E.; *et al.* [58]	1.72 (1.08-2.76)	Ser	African	6907/ 7644
Almeida, L.O.; *et al.* [59]	1.28 (0.03-2.10)	Ser		
Barnoud, T.; *et al.* [13]	+	Ser	African/ mice	
Jaiswal, P.K.; *et al.* [60]	-	Ser	Indian	200/ 200
Siraj, A.K.; *et al.* [61]	-	Ser	Saudi	223/ 229

Daugherty, C.L.; *et al.* [62]	-	Ser	Caucasian	191/<br>167
Ruggeri, R.M.; *et al.* [63]	-	Ser	Caucasian	79/30

Supplementary Table S3: Literature review table for Arg72Pro

**References**	**Association**	**AA (allele)**	**Population**	**Case/ Controls**
Elsaid, A.; *et al.* [64]	+	Pro	Egyptian	120/140
Sameer, A.S.; *et al.* [65]	+	Pro	Kashmiri	86/160
Pinto, G.R.; *et al.* [57]	-	Pro	Brazil	94/100
Santos, L.E.; *et al.* [66]	-	Pro	Brazil	94/159
Alawadi, S.; *et al.* [67]	0.166 (0.067-0.411) (decreased cancer risk)	Pro/Pro	Arab	288/188
Almeida, L.O.; *et al.* [59]	3.23 (1.71-6.08)	Pro		
Barnoud, T.; *et al.* [13]	+	Pro		
Mostaid, M.S.; *et al.* [68]	2.51 (1.38-4.82) and 4.62 (2.31-9.52)	Arg/Pro and Pro/Pro	Bangladesh	106/<br>116
Siraj, A.K.; *et al.* [61]	-	Pro	Saudi	223/<br>229
Daugherty, C.L.; *et al.* [62]	(1.16-2.82)	Arg/Arg	Caucasian	191/167
Ruggeri, R.M.; *et al.* [63]	p = 0.039 and p = 0.042	Pro/Arg (G/C) and Arg/Arg (G/G)	Caucasian	79/30
Nairuz, T.; *et al.* [69]	-		Bangladeshi	180/200
Diakite, B.; *et al.* [37]	+ : dominant model (DM)(1.09 (1.02-1.16));<br>additive model (AM)(1.09 (1.01-1.17))<br>- recessive model (RM)(1.07 (.97-1.18))<br>Pro: + association w/ CA for DM and AM; + association w/ AA for RM and AM	Pro	CA and AA	7841/<br>8876
Fang, Y.; *et al.* [70]	-	Pro	Chinese	373/<br>762
Drokow, E.K.; *et al.* [71]	+ : RM (1.276 (1.102-1.476));<br>DM (.891 (.802-.988))<br>+ : Chinese, American, African, Japanese, Indian pops	Pro	Muti-ethnic	2337/<br>9494

Supplementary Table S4: Allele frequency of rs1042522 and rs1800371 SNPs in *TP53* gene region

Chr	Base Pair	rs ID	Gene	Region	Ref Allele	Alt Allele	VAF	VAF_AA	VAF_CA
17	7676154	rs1042522	TP53	Exonic	G	C	0.4951	0.4127	0.6789
17	7676230	rs1800371	TP53	Exonic	G	A	0.0097	0.0141	0

Supplementary Table S5: Association of *TP53* SNPs with pathological upgrading from Diagnostic Biopsy to Radical Prostatectomy

Chrom	Location	rsID	Gene	Variant Type	Wild Allele	Variant Allele
chr17	chr17:7670065:C:T	rs59098034	TP53	intronic	C	T
chr17	chr17:7673523:A:G	rs1800899	TP53	intronic	A	G
chr17	chr17:7666224:G:A	rs74351250	ATP1B2\x3bTP53	intergenic	G	A
chr17	chr17:7669404:C:T	rs16956880	TP53	UTR3	C	T
chr17	chr17:7676230:G:A	rs1800371	TP53	exonic	G	A
chr17	chr17:7664122:T:C	rs150423780	ATP1B2\x3bTP53	intergenic	T	C
chr17	chr17:7666850:G:A	rs17882212	ATP1B2\x3bTP53	intergenic	G	A
chr17	chr17:7671546:C:T	rs9891744	TP53	intronic	C	T
chr17	chr17:7675353:C:T	rs35850753	TP53	UTR5	C	T
chr17	chr17:7671461:C:T	rs1641548	TP53	intronic	C	T
chr17	chr17:7673030:C:T	rs75732100	TP53	intronic	C	T
chr17	chr17:7665607:C:T	rs55745760	ATP1B2\x3bTP53	intergenic	C	T
chr17	chr17:7674797:T:C	rs1625895	TP53	intronic	T	C
chr17	chr17:7671403:C:T	rs17879377	TP53	intronic	C	T
chr17	chr17:7675361:A:G	rs9895829	TP53	UTR5	A	G
chr17	chr17:7667762:A:G	rs9894946	TP53	downstream	A	G
chr17	chr17:7674089:A:C	rs12951053	TP53	intronic	A	C
chr17	chr17:7674109:G:A	rs12947788	TP53	intronic	G	A
chr17	chr17:7672246:T:C	rs1642793	TP53	intronic	T	C
chr17	chr17:7674525:A:AT	rs539331761	TP53	intronic	A	AT
chr17	chr17:7667612:A:G	rs4968186	TP53	downstream	A	G
chr17	chr17:7673183:G:A	rs77697176	TP53	intronic	G	A
chr17	chr17:7671457:C:T	rs1641549	TP53	intronic	C	T
chr17	chr17:7671618:C:T	rs858528	TP53	intronic	C	T
chr17	chr17:7676734:C:T	rs8079544	TP53	intronic	C	T
chr17	chr17:7670066:G:A	rs112847074	TP53	intronic	G	A
chr17	chr17:7664734:T:C	rs60706951	ATP1B2\x3bTP53	intergenic	T	C
chr17	chr17:7666806:G:A	rs17881402	ATP1B2\x3bTP53	intergenic	G	A
chr17	chr17:7666998:A:G	rs1788451	ATP1B2\x3bT	intergenic	A	G

		2	P53			
chr17	chr17:7675327:C:T	rs2909430	TP53	UTR5	C	T
chr17	chr17:7676301:G:T	rs17883323	TP53	intronic	G	T
chr17	chr17:7667611:C:T	rs4968204	TP53	downstream	C	T
chr17	chr17:7676483:G:C	rs1642785	TP53	UTR5	G	C
chr17	chr17:7676154:G:C	rs1042522	TP53	exonic	G	C
chr17	chr17:7672415:A:G	rs12949655	TP53	intronic	A	G
chr17	chr17:7677182:C:T	rs12944939	TP53	intronic	C	T
chr17	chr17:7672395:A:T	rs111537395	TP53	intronic	A	T
chr17	chr17:7662815:A:G	rs12600850	ATP1B2\x3bTP53	intergenic	A	G
chr17	chr17:7669911:C:T	rs6503048	TP53	intronic	C	T
chr17	chr17:7666656:C:T	rs17881045	ATP1B2\x3bTP53	intergenic	C	T
chr17	chr17:7665125:G:A	rs140032142	ATP1B2\x3bTP53	intergenic	G	A
chr17	chr17:7662363:A:G	rs78496430	ATP1B2\x3bTP53	intergenic	A	G
chr17	chr17:7663627:GTTTA:G	rs200145124	ATP1B2\x3bTP53	intergenic	GTTTA	G
chr17	chr17:7676278:G:A	rs35117667	TP53	intronic	G	A
chr17	chr17:7668783:C:T	rs17884306	TP53	UTR3	C	T
chr17	chr17:7667660:T:C	rs17886760	TP53	downstream	T	C
chr17	chr17:7675519:A:G	rs1794287	TP53	intronic	A	G
chr17	chr17:7665009:C:T	rs72829457	ATP1B2\x3bTP53	intergenic	C	T
chr17	chr17:7662956:T:G	rs62062581	ATP1B2\x3bTP53	intergenic	T	G
chr17	chr17:7663008:CT:C	rs78337160	ATP1B2\x3bTP53	intergenic	CT	C
chr17	chr17:7667560:G:A	rs12949853	TP53	downstream	G	A
chr17	chr17:7672958:T:A	rs60699444	TP53	intronic	T	A
chr17	chr17:7672196:C:T	rs146137066	TP53	intronic	C	T
chr17	chr17:7663783:G:A	rs9674869	ATP1B2\x3bTP53	intergenic	G	A
chr17	chr17:7667505:G:C	rs9914052	TP53	downstream	G	C
chr17	chr17:7666380:A:C	rs8073498	ATP1B2\x3bTP53	intergenic	A	C
chr17	chr17:7666871:T:C	rs9893249	ATP1B2\x3bTP53	intergenic	T	C
chr17	chr17:7668134:G:A	rs1614984	TP53	downstrea	G	A

				m		
chr17	chr17:7663661:A:G	rs9674772	ATP1B2\x3bTP53	intergenic	A	G
chr17	chr17:7676963:G:A	rs9894227	TP53	intronic	G	A
chr17	chr17:7666228:C:G	rs144366923	ATP1B2\x3bTP53	intergenic	C	G
chr17	chr17:7667908:C:A	rs183153328	TP53	downstream	C	A
chr17	chr17:7669038:AAC:A	rs796594346	TP53	UTR3	AAC	A
chr17	chr17:7661841:ACCT:A	rs144289006	ATP1B2\x3bTP53	intergenic	ACCT	A
chr17	chr17:7663543:AAAAAG:A	rs368098733	ATP1B2\x3bTP53	intergenic	AAAAAG	A
chr17	chr17:7664197:C:T	rs35119871	ATP1B2\x3bTP53	intergenic	C	T
chr17	chr17:7674639:C:A	rs8069054	TP53	intronic	C	A
chr17	chr17:7667551:T:C	rs17881556	TP53	downstream	T	C
chr17	chr17:7667638:G:A	rs17883048	TP53	downstream	G	A
chr17	chr17:7674326:C:G	rs17880604	TP53	intronic	C	G
chr17	chr17:7668539:G:A	rs114831472	TP53	UTR3	G	A
chr17	chr17:7667753:T:C	rs17887200	TP53	downstream	T	C
chr17	chr17:7664709:C:T	rs147161837	ATP1B2\x3bTP53	intergenic	C	T
chr17	chr17:7670997:G:C	rs17883852	TP53	intronic	G	C
